# *Lacticaseibacilli* attenuated fecal dysbiosis and metabolome changes in *Candida*-administered bilateral nephrectomy mice

**DOI:** 10.3389/fimmu.2023.1131447

**Published:** 2023-03-09

**Authors:** Wiwat Chancharoenthana, Supitcha Kamolratanakul, Peerapat Visitchanakun, Supistha Sontidejkul, Thanya Cheibchalard, Naraporn Somboonna, Sarn Settachaimongkon, Asada Leelahavanichkul

**Affiliations:** ^1^ Department of Clinical Tropical Medicine, Faculty of Tropical Medicine, Mahidol University, Bangkok, Thailand; ^2^ Tropical Immunology and Translational Research Unit (TITRU), Department of Clinical Tropical Medicine, Faculty of Tropical Medicine, Mahidol University, Bangkok, Thailand; ^3^ Center of Excellence on Translational Research in Inflammatory and Immunology (CETRII), Department of Microbiology, Chulalongkorn University, Bangkok, Thailand; ^4^ Program in Biotechnology, Faculty of Science, Chulalongkorn University, Bangkok, Thailand; ^5^ Department of Microbiology, Faculty of Science, Chulalongkorn University, Bangkok, Thailand; ^6^ Microbiome Research Unit for Probiotics in Food and Cosmetics, Chulalongkorn University, Bangkok, Thailand; ^7^ Department of Food Technology, Faculty of Science, Chulalongkorn University, Bangkok, Thailand

**Keywords:** uremia, microbiome, short-chain fatty acid, fungi, kidney

## Abstract

The impacts of metabolomic changes (reduced short-chain-fatty acids; SCFAs) in uremic condition is not fully understood. Once daily *Candida* gavage with or without probiotics (different times of administration) for 1 week prior to bilateral nephrectomy (Bil Nep) in 8-week-old C57BL6 mice as the possible models more resemble human conditions were performed. *Candida*-administered Bil Nep mice demonstrated more severe conditions than Bil Nep alone as indicated by mortality (n = 10/group) and other 48 h parameters (n = 6-8/group), including serum cytokines, leaky gut (FITC-dextran assay, endotoxemia, serum beta-glucan, and loss of Zona-occludens-1), and dysbiosis (increased *Enterobacteriaceae* with decreased diversity in microbiome analysis) (n = 3/group for fecal microbiome) without the difference in uremia (serum creatinine). With nuclear magnetic resonance metabolome analysis (n = 3-5/group), Bil Nep reduced fecal butyric (and propionic) acid and blood 3-hydroxy butyrate compared with sham and *Candida*-Bil Nep altered metabolomic patterns compared with Bil Nep alone. Then, *Lacticaseibacillus rhamnosus* dfa1 (SCFA-producing *Lacticaseibacilli*) (n = 8/group) attenuated the model severity (mortality, leaky gut, serum cytokines, and increased fecal butyrate) of Bil Nep mice (n = 6/group) (regardless of *Candida*). In enterocytes (Caco-2 cells), butyrate attenuated injury induced by indoxyl sulfate (a gut-derived uremic toxin) as indicated by transepithelial electrical resistance, supernatant IL-8, *NFκB* expression, and cell energy status (mitochondria and glycolysis activities by extracellular flux analysis). In conclusion, the reduced butyrate by uremia was not enhanced by *Candida* administration; however, the presence of *Candida* in the gut induced a leaky gut that was attenuated by SCFA-producing probiotics. Our data support the use of probiotics in uremia.

## 1 Introduction

Uremia is an accumulation of toxins in the blood due to the loss of renal function from both chronic and acute kidney injury that are common health-care problems worldwide (1). With an inadequate excretion of uremic toxins through the urine, several water-soluble toxins are excreted into the intestine as an alternative route causing an alteration in the intestinal environments that select the growth of some organisms (gut dysbiosis) (2). As such, the hydrolysis of urea (a major uremic toxin) by urease-producing bacteria and the digestion of other toxins facilitates the survival of some microbes (3–5). Indeed, uremia from both chronic kidney disease (CKD) and acute kidney injury (AKI) induced gut dysbiosis (6–8) and the dysbiosis also worsens uremic complications through several mechanisms (9). Accordingly, gut bacteria during uremia-induced dysbiosis facilitated the production of gut-derived uremic toxins, such as p-cresol, indoxyl sulfate, and trimethylamine N-oxide, which enhanced damage to endothelium, kidney, heart, and intestine through chronic inflammation, oxidative stress, and atherosclerosis (10, 11). Additionally, uremic toxins have a direct impact on the enterocytes resulting in enterocytic cell death (2) and also reduce short-chain-fatty-acid (SCFA)-producing bacteria (12). All of these factors from uremia induce defects of gut permeability (or gut barrier) allowing the translocation of pathogen molecules from the gut into the blood circulation (gut leakage or leaky gut). Indeed, the presence of endotoxin, a major cell wall component of Gram-negative bacteria (the most abundance gut organisms), and (1➔3)-β-D-glucan (BG), the main molecule in the cell wall of fungi (the second most abundance gut microbes), in the blood of patients with uremia are demonstrated as a proof of concept for uremia-induced leaky gut (13, 14). During uremia, the presence of these pathogen molecules in blood further enhances uremic toxin-induced chronic inflammatory responses, especially with the induction of innate immunity against microbial molecules that foreign to the host (15). Hence, uremia induces gut dysbiosis and chronic inflammation through impacts of uremia on gut microbes (selection of some bacteria) and host cells (cell injury by oxidative stress from the toxins), then the dysbiosis causes a more severe systemic inflammation with renal function worsening through the leaky gut. However, impacts of uremia on gut dysbiosis in the current literature are mostly focusing on gut bacteria, although gut fungi, especially *Candida albicans* in human intestines, are the second most abundance microbes in the gut that demonstrate some interaction with gut bacteria and enterocytes (16). Despite the larger size of fungi (10–12 µm of *Candida* in a yeast form) than bacteria (0.5–2 µm), the fungal abundance in feces by gene copies using 18S rRNA is 1,000-fold lower than 16S rRNA of bacteria with approximately 267 fungal species compared with more than 3,500 bacterial species in the gut (17). The bacterial community varies in quantity and composition from the stomach to the colon (10^2^ versus 10^11^ cells/gram feces in the stomach and colon, respectively), whereas fungi seem to be localized mostly in the colon, with an average of 10^6^ fungal cells per gram of colon content (18). Interestingly, the presence of gut fungi selectively induces the growth of some gut bacteria (dysbiosis), partly due to i) a digestion ability toward BG of fungal cell wall (glucanase enzymes) as mixing BG into the culture medium enhances the growth of some bacteria (19, 20), and ii) the bacterial tolerance against *Candida* toxins (21). Then, fungi in the gut can alter bacterial compositions in the gut and contribute to leaky gut-induced systemic inflammation in uremia through glucanemia and endotoxemia (22) and the abundance of gut organisms might be correlated with the level of pathogen molecules in blood during uremia-induced leaky gut (23). Due to the difference in the abundance of fungi in rodents versus humans, *Candida* administration might make the models more resemble humans (19, 24–26). Indeed, the abundance of *Candida* spp. in mouse feces is not high enough to be detectable by stool culture (27), which is different from cultures of human feces (28). Unfortunately, impacts of gut fungi, especially *C. albicans*, in several conditions with leaky gut is not properly considered different from gut bacteria, partly because gut fungi do not seem to cause illness directly. Although we previously demonstrated an impact of oral *Candida* administration in acute and chronic uremia through bilateral nephrectomy and 5/6 nephrectomy models (2, 12, 29, 30), respectively, the impact of gut fungi on metabolome analysis has never been described. As such, SCFAs (acetate, propionate, and butyrate) are metabolic products of anaerobic bacterial fermentation, especially on the complex carbohydrates-rich diets, in the intestine that is important for the maintenance of intestinal homeostasis (31–33). Due to the possible depletion of SCFAs by uremic toxin-induced dysbiosis, the administration of probiotics, the health beneficial organisms used in several situations, might attenuate leaky gut and systemic inflammation in several conditions, including uremia (34, 35). Although the benefits of probiotics in uremic conditions (acute and chronic kidney injury) are mentioned (7), the exploration of SCFAs and metabolome in uremia is still less. Hence, our objective was to explore the impact of gut *Candida* on the metabolome changes, especially SCFAs, in feces and in blood during acute uremia and also tested the effectiveness of *Lacticaseibacillus rhamnosus* dfa1 which are the recently isolated SCFA-producing *Lacticaseibacilli* from the Thai healthy volunteers from our previous study (36). We hypothesized that SCFAs from probiotics themselves or from other probiotic-promoted bacteria might attenuate uremia-induced intestinal damage and tested the hypothesis *in vivo* and *in vitro*.

## 2 Materials and methods

### 2.1 Animals

Male 8-week-old C57BL/6 mice from Nomura Siam International (Pathumwan, Bangkok, Thailand) were used according to the approval by the Institutional Animal Care and Use Committee of the Faculty of Medicine, Chulalongkorn University, Bangkok, Thailand, following the animal care and use protocol U.S. National Institutes of Health (NIH). *Candida*-administered bilateral nephrectomy (Bil Nep) or Bil Nep alone, as previously described, were conducted to test the possible impact of gut fungi. Briefly, *Candida albicans* from the American Type Culture Collection (ATCC 90028) (Fisher Scientific, Waltham, MA, USA) prepared in Sabouraud dextrose broth (SDB) (Oxoid, Hampshire, UK) at 1 × 10^6^ CFU in a 0.3-mL phosphate buffer solution (PBS), or PBS alone, following by *Lacticaseibacillus rhamnosus* dfa1 that isolated from Thai population (Chulalongkorn University, Bangkok, Thailand) (36) at 1 × 10^8^ CFU in 0.3 mL PBS, or PBS alone, were orally administered at 8 and 12 a.m., respectively, for 7 days prior to Bil Nep surgery. Subsequently, Bil Nep was performed 6 h after the last oral gavage (approximately at 6 p.m.) through abdominal incision according to previous publications (37–39). In the sham group, renal vessels and ureters were only identified before closing the abdominal incision, and fentanyl at 0.03 mg/kg of body weight in 0.5 mL normal saline solution (NSS) was subcutaneously injected after the operation for both analgesia and fluid replacement. Notably, the *L. rhamnosus* were cultured on de Man-Rogosa-Sharpe (MRS) agar (Oxoid) under anaerobic conditions with gas generation sachets (AnaeroPack-Anaero; Mitsubishi Gas Chemical Co., Inc., Japan) at 37°C for 48 h before quantitative preparation by the determination of the optical density at 600 nm (OD600) as previously described (2). There were 6 mice per group in sham and 8 mice per group for Bil Nep alone and *Candida*-administered Bil Nep. For probiotics experiments, there were 6 mice per group for sham, Bil Nep, and probiotic-administered Bil Nep, while there were 8 mice per group for *Candida*-administered Bil Nep with and without probiotics. In the non-survival experiments, mice were sacrificed at 48 h post-surgery under isoflurane anesthesia (with blood and colon collection). Survival analysis was performed using other groups of mice with 10 mice per group and the moribund mice were humanly sacrificed. Colons, 2 cm distal to caecum, were put in tissue frozen in optimal cutting temperature (OCT) compound (Tissue-Tek OCT compound; Sakura Finetek USA, Inc., Torrance, CA, USA) for fluorescent microscopic evaluation. Feces from all parts of the colon were combined and collected for fecal microbiome analysis. Of note, the data at 0 h were collected 3 days before the operation and were used as the baseline values.

### 2.2 Mouse sample analysis

Serum creatinine (renal injury) and serum cytokines were evaluated by QuantiChrom creatinine colorimetric assay (DICT-500) (Bioassay, Hayward, CA, USA) and enzyme-linked immunosorbent assay (ELISA) (Invitrogen, Carlsbad, CA, USA), respectively. Gut leakage was determined by i) the detection of fluorescein isothiocyanate-dextran (FITC-dextran), an intestinal nonabsorbable molecule in serum, after an oral administration, ii) serum endotoxin (LPS, a major cell wall components of Gram-negative bacteria), iii) bacteremia, and iv) staining of Zona occludens-1 (ZO-1, an enterocyte tight junction molecule) as previously described (2). For FITC-dextran assays, 0.5 mL of FITC-dextran (molecular weight, 4.4 kDa) (Sigma-Aldrich) at 25 mg/mL was orally administered 3 h prior to blood collection at sacrifice before analysis by fluorescence spectroscopy (Varioskan Flash; Thermo Scientific) at excitation and emission wavelengths of 485 and 528 nm, respectively, with a standard curve of FITC-dextran. Serum LPS was determined by HEK-Blue LPS detection (InvivoGen, San Diego, CA, USA) and bacterial burdens were evaluated by adding 25 µL of blood samples into blood agar for 24 h incubation at 37°C before colony enumeration. For ZO-1 determination, the colons in the 5-µm-thick-frozen sections were stained with a primary antibody against ZO-1 (61-7300) (Thermo Fisher Scientific) (1:200) followed by the secondary antibody Alexa Fluor 546 goat anti-rabbit IgG (A-11035) (Life Technologies, USA) (1:200) and 4’,6-diamidino-2-phenylindole (DAPI; BioLegend, USA) (1:1,000) (nucleus staining color) before visualization and analyzed with a Zeiss LSM 800 confocal microscope (Carl Zeiss, USA) following a previous publication (2).

### 2.3 Fecal microbiome analysis

Fecal microbiota analysis was performed according to methods reported in previous publications (26, 40) using the total DNA from feces of individual mice. Mouse feces were collected by placing mice in metabolic cages (Hatteras Instruments, Cary, NC, USA) for a few hours before the collection of feces (0.25 g) from each mouse in different cages for microbiome analysis to avoid the influence of allocoprophagy (a habit of mice that ingest feces from other mice). The fecal microbiota analysis was performed in 3 fecal samples from 3 mice per group of sham, Bil Nep alone, and Bil Nep with *Candida*. Briefly, a power DNA isolation kit (MoBio, Carlsbad, CA, USA), metagenomic DNA quality determination (agarose gel electrophoresis and nanodrop spectrophotometry), universal prokaryotic primers; forward 515F (5’-GTGCCAGCMGCCGCGGTAA-3’) and reverse primer 806R (5’-GGACTACHVGGGTWTCTAAT-3’), and 16S rRNA V4 library (appended 50 Illumina adapter and 30 Golay barcode sequences) were used. Each sample (240 ng) was applied to the MiSeq300 sequencing platform (Illumina, San Diego, CA, USA) with Mothur’s standard quality screening operating procedures in MiSeq platform with aligned and assigned taxa (operational taxonomic units [OTUs]) based on default parameters were used (2).

### 2.4 Metabolome analysis in plasma and fecal samples

Sample preparation and metabolome analysis by nuclear magnetic resonance (NMR) spectroscopy following previous publications was conducted (41, 42). Briefly, plasma (400 µL) or feces (0.2 g) at pH of 7.5 in 2.4 mL ultrapure water was vortexed for 10 minutes, centrifuged at 14,000 ×g for 10 minutes at 4°C before transferring the supernatant (500 μL) into an Eppendorf tube for filtering through a Pall Nanosep^®^ (3 kDa molecular weight) (Pall life science, Ann Arbor, MI, USA). Then, the filtrate was mixed 1:1 (vol./vol.) with the buffer, which consisted of 300 mM KH2PO4, 10% (w/w) deuterium oxide (D2O), and 1 mM 3‐(Trimethylsilyl) propionic‐2, 2, 3, 3‐d4 acid sodium salt (TSP) at pH 7.5, as the internal standard (41). Additionally, NOESY (nuclear overhauser enhancement spectroscopy) 1D‐1H‐NMR measurements were performed in a 500 MHz NMR spectrometer (Bruker, Rheinstetten, Germany); the 1H NMR spectra were aligned and calibrated based on the internal standard (TSP) peak. For each spectrum, chemical shift (δ) across a range of 0.00-10.00 ppm was segmented (binning) with an interval of 0.02 ppm and the signal intensity in each bin was integrated using Topspin (V 4.0.7, Bruker Biospin) to derive a quantity of each spectrum. The identification of each spectrum (metabolite) was assigned according to the ChenomxNMR suite 8.5 library (Chenomx Inc., Alberta, Canada). The MetaboAnalyst 5.0 (http://www.metaboanalyst.ca/) was used for metabolome data normalization (i.e., by sample median and auto-scaled by mean-centering and dividing by the standard deviation of each variable), clustering algorithm by Ward’s method, and statistical analyses (43). Data visualizations were performed using GraphPad Prism version 8.0 software (GraphPad, La Jolla, CA, USA) and MetaboAnalyst 5.0. There were 5 samples (from 5 mice) in sham and 3 samples (from 3 mice) in Bil Nep and *Candida*-administered Bil Nep for fecal metabolome analysis. Meanwhile, there were 4 samples (from 4 mice) in sham, 3 samples (from 3 mice) in Bil Nep, and 4 samples (from 4 mice) of *Candida*-administered Bil Nep for blood metabolome analysis.

### 2.5 Short chain fatty acid evaluation

Both *L. rhamnosus* dfa1 and *Enterococcus faecium* dfa1 were prepared in MRS media as mentioned above, while *Bifidobacterium longum* dfa1 was cultured in the brain heart infusion (BHI) broth (Oxoid) according to the previous publications (36, 44). Then, short chain fatty acids (SCFAs) in the condition media were analyzed by gas chromatography–mass spectrometry (GC-MS) using the headspace solid-phase microextraction method with an Agilent 6890 GC equipped with an Agilent 5973 mass selective detector (Agilent Technologies) according to a previous publication (45). Briefly, the dimension of the column was 0.25 mm×30 m×0.25 μm with Helium carrier gas at 13.7 ml/min. The temperature program was 10 min isothermal at 50°C, 10 min rising to 240°C with 15°C/min. The injection port temperature is 200°C while the detector port temperature is 250°C. The mass spectrometer was operated in the electron impact mode at 70 eV with a scan range was 40–200 amu. A standard curve was obtained for the calculation of each SCFA concentration. For fecal SCFA, the fecal fatty acids were extracted before the determination by GC-MS according to a previous publication (46). In brief, feces (20 mg in 500 µL of NSS) were added with 10% H2SO4 before fatty acids separation by anhydrous ether (800 μL) and centrifuged (18,000 g for 15 min). Then, the upper ether phase was mixed with 0.25 g of anhydrous Na2SO4 for 30 min, centrifuged (18,000 g for 5 min), and SCFAs in the upper diethyl ether phase were determined by GC-MS as mentioned above.

### 2.6 The *in vitro* experiments on Caco-2 cells

The influence of indoxyl sulfate, a gut-derived uremia toxin, and butyrate, a well-known SCFA, in the enterocytes was examined using the Caco-2 cell line as previously described (12). As such, the Caco‐2 (ATCCHTB-37) (American Type Culture Collection, Manassas, VA, USA) at 2 × 10^6^ cells/well in Dulbecco’s Modified Eagle Medium (DMEM) were incubated with the different concentrations of indoxyl sulfate (Sigma‐Aldrich, St. Louis, MO, USA) for 24 h before the determination of cell viability with the 2 h incubation (at 37°C in the dark) by 0.5 mg/mL of tetrazolium dye 3‐(4,5‐dimethylthiazol‐2‐yl) ‐2,5‐diphenyltetrazolium (MTT) solution (Thermo Fisher Scientific). The MTT assay is a colorimetric assay for measuring cell metabolic activity based on the ability of nicotinamide adenine dinucleotide phosphate (NADPH)-dependent cellular oxidoreductase enzymes to reduce the MTT tetrazolium dye into the insoluble purple color formazan. After the incubation, the MTT solution was removed and diluted with dimethyl sulfoxide (DMSO) (Thermo Fisher Scientific) before measurement with a Varioskan Flash microplate reader at an absorbance of optical density at 570 nm. On the other hand, Caco‐2 cells at 5 × 10^4^ cells per well were seeded onto the upper compartment of 24‐well Boyden chamber trans wells (Sigma‐Aldrich), using high glucose DMEM supplemented with 20% Fetal Bovine Serum (FBS), 1% HEPES, 1% sodium pyruvate, and 1.3% Penicillin/Streptomycin for 15 days to establish the monolayer of the cells before 24 h incubation with indoxyl sulfate (Sigma‐Aldrich) (0.5 mM) alone or with butyrate (Sigma‐Aldrich) at the indicated concentrations. After that, the transepithelial electrical resistance (TEER) was measured as previously described (47) as demonstrate in ohm (Ω) × cm2 using the epithelial volt‐ohm meter (EVOM2™, World precision instruments, Sarasota, FL, USA) by placing electrodes in the supernatant at the basolateral chamber and in the apical chamber. The TEER values in media culture without Caco‐2 cells were used as a blank and were subtracted from all other measurements. In parallel, the supernatant cytokines were measured by ELISA (Invitrogen).

### 2.7 Extracellular flux analysis

To explore the impact of uremic toxin and butyrate on cell energy status, the extracellular flux analysis using the Seahorse XFp Analyzers (Agilent, Santa Clara, CA, USA) for the determination of mitochondrial activity and glycolysis through the oxygen consumption rate (OCR) and extracellular acidification rate (ECAR), respectively (25, 48–50). In the Seahorse XFp Analyzers, mitochondrial ATP production rates are determined by the rate of oxygen consumption (OCR) in the oxidative phosphorylation pathway that is needed by mitochondria and the ECAR is a result of lactate production in the glycolysis pathway that is used as a representative for glycolysis activity. Both OCR and ECAR are simultaneously measured in real-time in culture well plates using fluorescent sensors in the machine analyzer. As such, Caco-2 cells (1 × 10^4^ cells/well) were grown in modified DMEM, with 0.5 mM of indoxyl sulfate (a gut-derived uremic toxin) with or without 1 mM butyrate (a representative SCFA) for 24 h in the Seahorse cell culture plate before replacing by Seahorse substrates (glucose, pyruvate, and L-glutamine) (Agilent, 103575–100) in pH 7.4 at 37˚C for 1 h prior to the challenge with different metabolic interference compounds for mitochondrial reactions, including oligomycin, carbonyl cyanide-4-(trifluoromethoxy)-phenylhydrazone (FCCP), and rotenone/antimycin A, and for glycolysis intervention, including glucose, oligomycin, and 2-Deoxy-d-glucose (2-DG), according to the manufacturer’s instructions. Data from Seahorse Wave 2.6 software were also conducted based on the following equations: maximal respiration = (OCR between FCCP and rotenone/antimycin A) – (OCR after rotenone/antimycin A); respiratory reserve = (OCR between FCCP and rotenone/antimycin A) – (OCR before oligomycin); and glycolysis = ECAR between glucose and oligomycin.

### 2.8 Statistical analysis

Mean ± standard error (SE) was used for data presentation. The differences between groups were examined for statistical significance by one-way analysis of variance (ANOVA) followed by Tukey’s analysis or Student’s *t-*test for comparisons of multiple groups or 2 groups, respectively. All statistical analyses were performed with SPSS 11.5 software (SPSS, IL, USA) and Graph Pad Prism version 7.0 software (La Jolla, CA, USA). A *p*-value of < 0.05 was considered statistically significant.

## 3 Results

### 3.1 *Candida* administration worsened bilateral nephrectomy mice through dysbiosis-induced leaky gut that enhanced systemic inflammation

**Figure 1:**
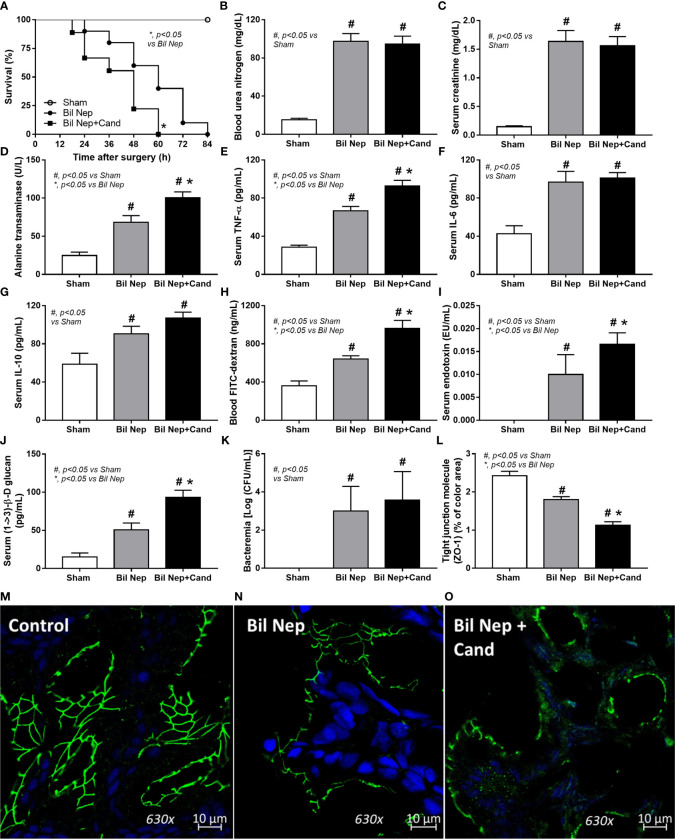
Characteristics of bilateral nephrectomy (Bil Nep) mice orally administered by normal saline solution (NSS) or Candida albicans (Bil Nep+Cand) or control sham surgery (Sham) as determined by survival analysis **(A)**, blood urea nitrogen **(B)**, serum creatinine **(C)**, serum cytokines (TNF-α, IL-6, and IL-10) **(D–G)**, and parameters of gut barrier defect, including FITC-dextran assay **(H)**, endotoxemia **(I)**, (1→3-β-D-glucan) **(J)**, bacteremia **(K)**, and the abundance of tight junction molecule (Zona occluden-1; ZO-1) **(L)** in the colon (percentage of the green fluorescent color) with the representative fluorescent-stained pictures (original magnification 630x) **(M–O)** are demonstrated (n = 10/group for **A** and 6-8/group for others).

To resemble human conditions, *C. albicans* was orally administered before bilateral nephrectomy (Bil Nep) surgery. As such, *Candida* worsened Bil Nep mice as indicated by mortality, liver injury (alanine transaminase), serum cytokines (TNF-α, IL-6, and IL-10), leaky gut by FITC-dextran assay, endotoxemia, serum (1➔3)-β-D-glucan (BG), and enterocyte tight junction protein (Zona occludens-1; ZO-1), but not renal injury (blood urea nitrogen and serum creatinine), and bacteremia, when compared with the control Bil Nep using only normal saline solution (NSS) gavage (**Figures 1A–O**). In sham mice, *Candida* did not alter any parameters when compared with sham control (data not shown). The increased endotoxin (LPS) and BG in serum (**Figures 1K, L**) along with enterocytes damage (ZO-1) (**Figures 1N, O**) of Bil Nep mice compared with sham mice indicated uremia-induced leaky gut which was more prominent in *Candida*-administered Bil Nep compared with Bil Nep alone.

**Figure 2:**
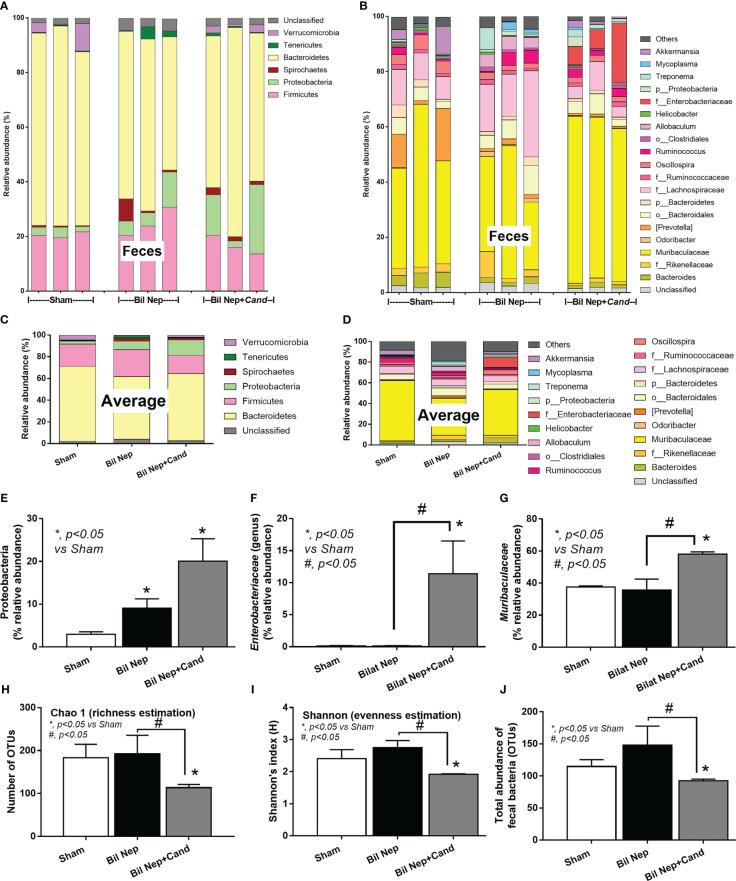
Fecal microbiota analysis of mice with sham, bilateral nephrectomy (Bil Nep), and Bil Nep with *Candida* administration at 48 h post-operation as indicated by the relative abundance of bacteria at the phylum and genus levels with the average value **(A–D)**, the abundances of some bacteria in graph presentation **(E–G)**, and the alpha diversity (Chao 1 and Shannon index) with a total abundance of bacteria in operational taxonomic units (OTUs) **(H–J)** are demonstrated.

In parallel, *Candida* also induced gut dysbiosis as indicated by fecal microbiome analysis at 48 h of experiments (**Figures 2A–D**). With the collection of fecal samples from 3 mice in each experimental group, there was an elevation in Proteobacteria (a major phylum of Gram-negative bacteria including pathogenic microbes), Enterobacteriaceae (a group of pathogenic Gram-negative bacilli), and Muribaculaceae (Gram-negative anaerobes in Bacteroides group) in *Candida*-administered Bil Nep when compared with Bil Nep alone (**Figures 2E–G**). Additionally, *Candida*-induced dysbiosis was also indicated by the reduction in total bacterial abundance and the diversity (Chao-1 and Shannon scores) when compared with Bil Nep alone (**Figures 2H–J**). Proteobacteria of Bil Nep mice were also more prominent than sham mice despite the non-difference in total bacteria abundance and diversity (**Figures 2A–J**), supporting uremia-induced gut dysbiosis. Hence, Bil Nep without *Candida* demonstrated uremia-induced leaky gut through an enhanced pathogenic bacteria (Proteobacteria) resulting in endotoxemia and glucanemia with systemic inflammation-induced liver injury and nearly all of these parameters (fecal Proteobacteria, leaky gut, endotoxemia, glucanemia, serum TNF-a, and liver injury) was worsened by *Candida* administration in Bil Nep compared with Bil Nep alone.

### 3.2 Altered metabolome characteristics in feces and in the blood of uremic mice with and without *Candida* administration

**Figure 3:**
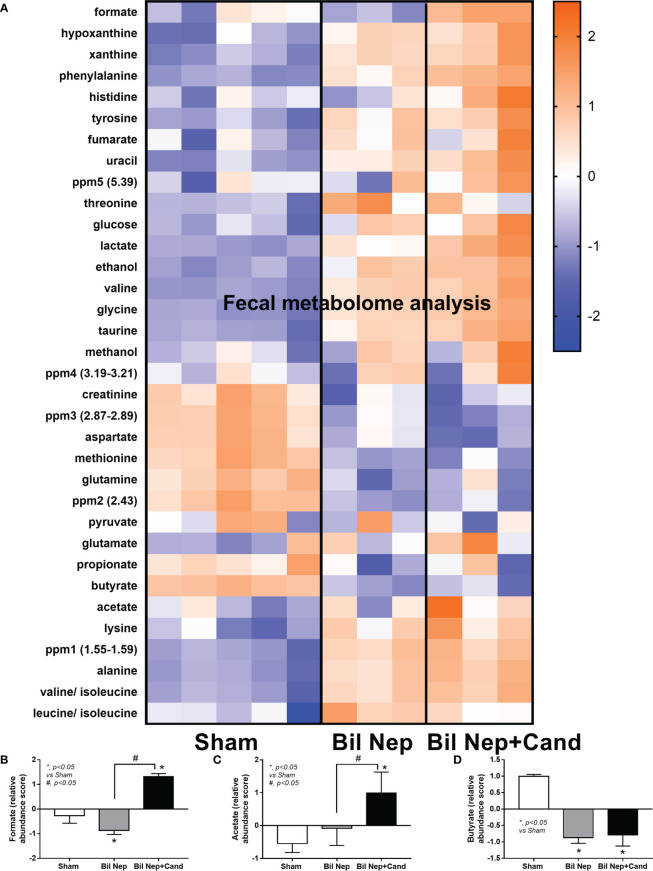
Fecal metabolome analysis of mice with sham, bilateral nephrectomy (Bil Nep), and Bil Nep with *Candida* administration at 48 h post-operation as indicated by the heat-map of the metabolites **(A)** with the graphs of some substances (formate, acetate, and butyrate) **(B–D)** are demonstrated. The color scale bars are demonstrated by log2 fold change. The average values from sham (5 samples) and Bil Nep with or without *Candida* (3 samples per group) are presented in the column graph for visualization of the comparison.

**Figure 4:**
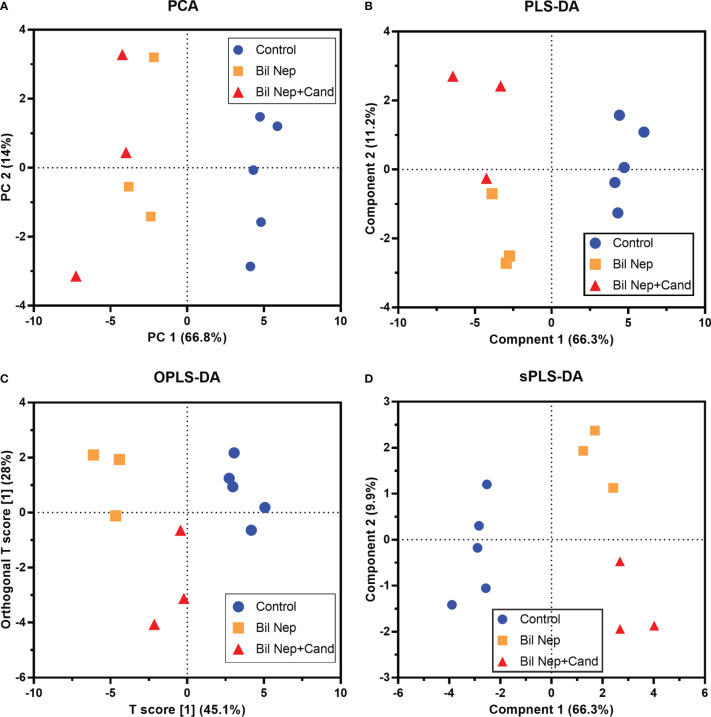
Fecal metabolome analysis of mice with sham, bilateral nephrectomy (Bil Nep), and Bil Nep with *Candida* administration at 48 h post-operation as indicated by several score plot analyses, including the relationships among plasma metabolome profiles by principal component analysis (PCA) **(A)**, orthogonal partial least squares-discriminant analysis (OPLS-DA) **(B)**, partial least squares-discriminant analysis (PLS-DA) **(C)**, and sparse partial least squares - discriminant analysis (sPLS-DA) **(D)** are demonstrated.

**Figure 5:**
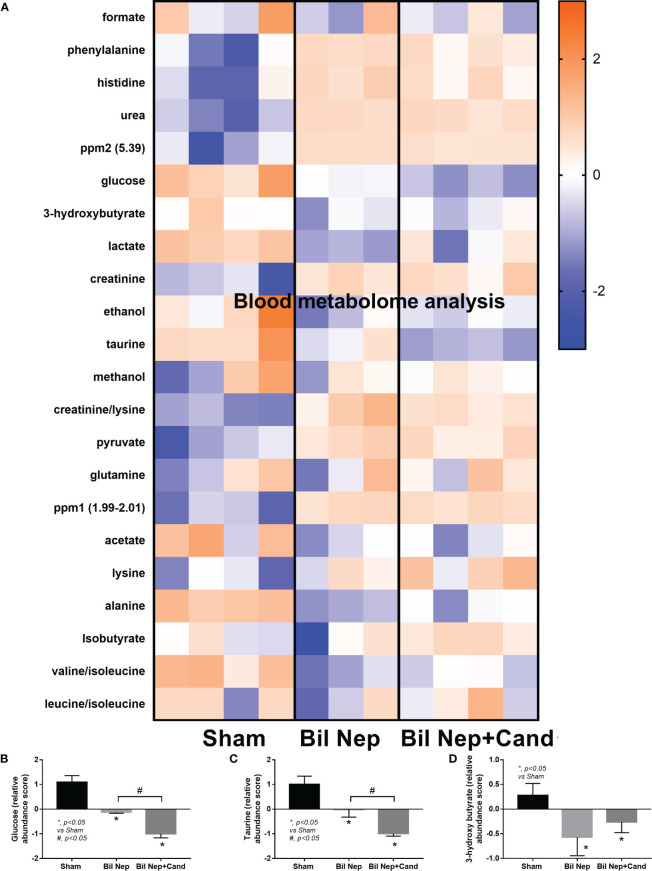
Blood metabolome analysis of mice with sham, bilateral nephrectomy (Bil Nep), and Bil Nep with *Candida* administration at 48 h post-operation as indicated by the heat-map of the metabolites **(A)** with the graphs of some substances (formate, acetate, and butyrate) **(B–D)** are demonstrated. The color scale bars are demonstrated by log2 fold change. The average values from sham (4 samples), Bil Nep (3 samples), and Bil Nep with *Candida* (3 samples) are presented in the column graph for visualization of the comparison.

**Figure 6:**
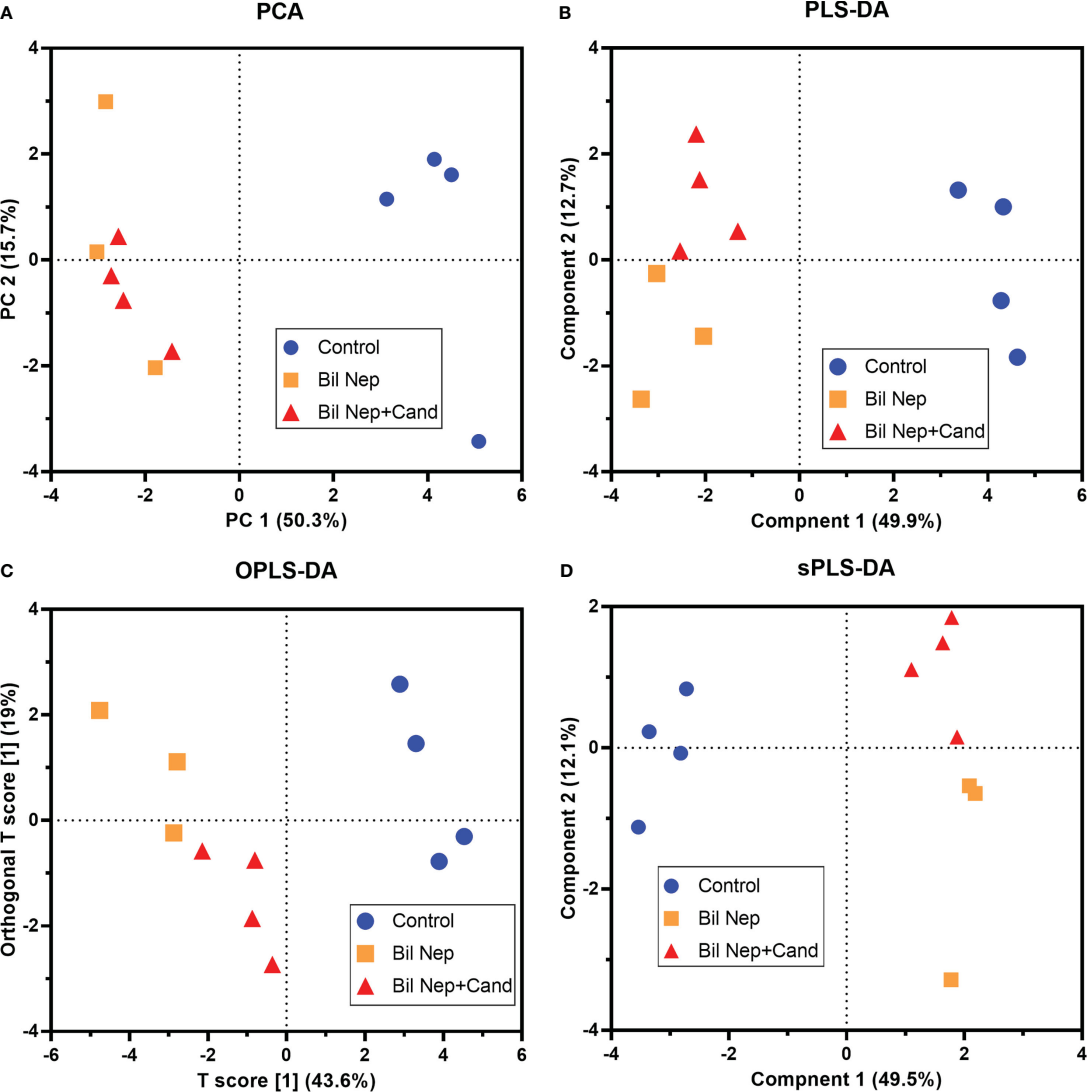
Blood metabolome analysis of mice with sham, bilateral nephrectomy (Bil Nep), and Bil Nep with *Candida* administration at 48 h post-operation as indicated by several score plot analyses, including the relationships among plasma metabolome profiles by principal component analysis (PCA) **(A)**, orthogonal partial least squares-discriminant analysis (OPLS-DA) **(B)**, partial least squares-discriminant analysis (PLS-DA) **(C)**, and sparse partial least squares - discriminant analysis (sPLS-DA) **(D)** are demonstrated.

The excretion of uremic toxins into the gut was not only inducing fecal dysbiosis (**Figures 2A–J**) but also altered the fecal metabolome (**Figures 3A, B**). In comparison with sham mice, Bil Nep mice demonstrated a significant increase in i) nitrogenous bases and derivatives (hypoxanthine, xanthine, and uracil), ii) amino acids (threonine, phenylalanine, lysine, valine, isoleucine, tyrosine, glycine, taurine, alanine, and leucine), iii) energy-related compounds (lactate, glucose, fumarate and ethanol) (**Figure 3A**). Meanwhile, Bil Nep induced a significant decrease in i) amino acids (lysine, aspartate, methionine, glutamine), ii) SCFAs (propionate and butyrate), and iii) an energy-related metabolite (pyruvate) when compared with sham feces (**Figure 3A**). The fecal metabolome of Bil Nep versus Candida-Bil Nep mice was similar except for the higher formate and acetate in the latter group (**Figures 3B–D**). Additionally, the separation between sham control versus Bil Nep feces and Bil Nep versus *Candida*-Bil Nep feces was demonstrated by several plot analyses, including principal component analysis (PCA; the data simplification into fewer summary dimensions while retaining trends and patterns) and partial least squares-discriminant analysis (PLS-DA; the data projection into the non-direct observed structure to find the fundamental relations between two matrices) with modified analyses, including orthogonal PLS-DA (OPLS-DA), and sparse PLS-DA (sPLS-DA) (**Figures 4A–D**). In parallel, blood metabolome in Bil Nep compared with sham demonstrated an increase in i) some amino acids (phenylalanine, lysine, and histidine), ii) uremic toxins (urea and creatinine), and iii) pyruvate, with a decrease in i) some amino acids (taurine, alanine, leucine, isoleucine, and valine), ii) energy-related compounds (glucose, lactate, and ethanol), and iii) SCFAs (3-hydroxybutyrate and acetate) (**Figures 5A–D**), supported by all plot analyses (**Figures 6A–D**). Meanwhile, the difference between Bil Nep versus *Candida*-Bil Nep was subtle (**Figures 5A, B**) as could be demonstrated only by PLS-DA and the modifications (OPLS-DA and sPLS-DA) (**Figures 6B–D**). Due to the importance of SCFAs in enterocyte homeostasis (18), the reduced butyrate (and propionate) in feces (**Figure 3A**) and 3-hydroxy butyrate in the blood (**Figure 5A**) of uremic mice, perhaps due to uremia-induced gut dysbiosis (**Figures 2A–J**), might partly be responsible for uremia-induced leaky gut (**Figures 1J–O**). In short, the uremia-induced metabolome alteration in feces and in blood of Bil Nep compared with sham mice from was clearly demonstrated, especially through the reduced butyrate (an important SCFA) in feces and in the blood; however, *Candida* administration did not worsen the decreased butyrate. These data implied that the worsened conditions of *Candida*-Bil Nep over Bil Nep alone (**Figures 1J–O**) were not due to reduced SCFA but possibly because of other adverse effects of the fungi, such as *Candida*-induced gut dysbiosis (**Figures 2A–J**) that causing a more severe leaky gut-induced systemic inflammation.

### 3.3 Probiotics attenuated uremia-induced leaky gut, partly through an impact of short-chain fatty acids on enterocytes

**Figure 7:**
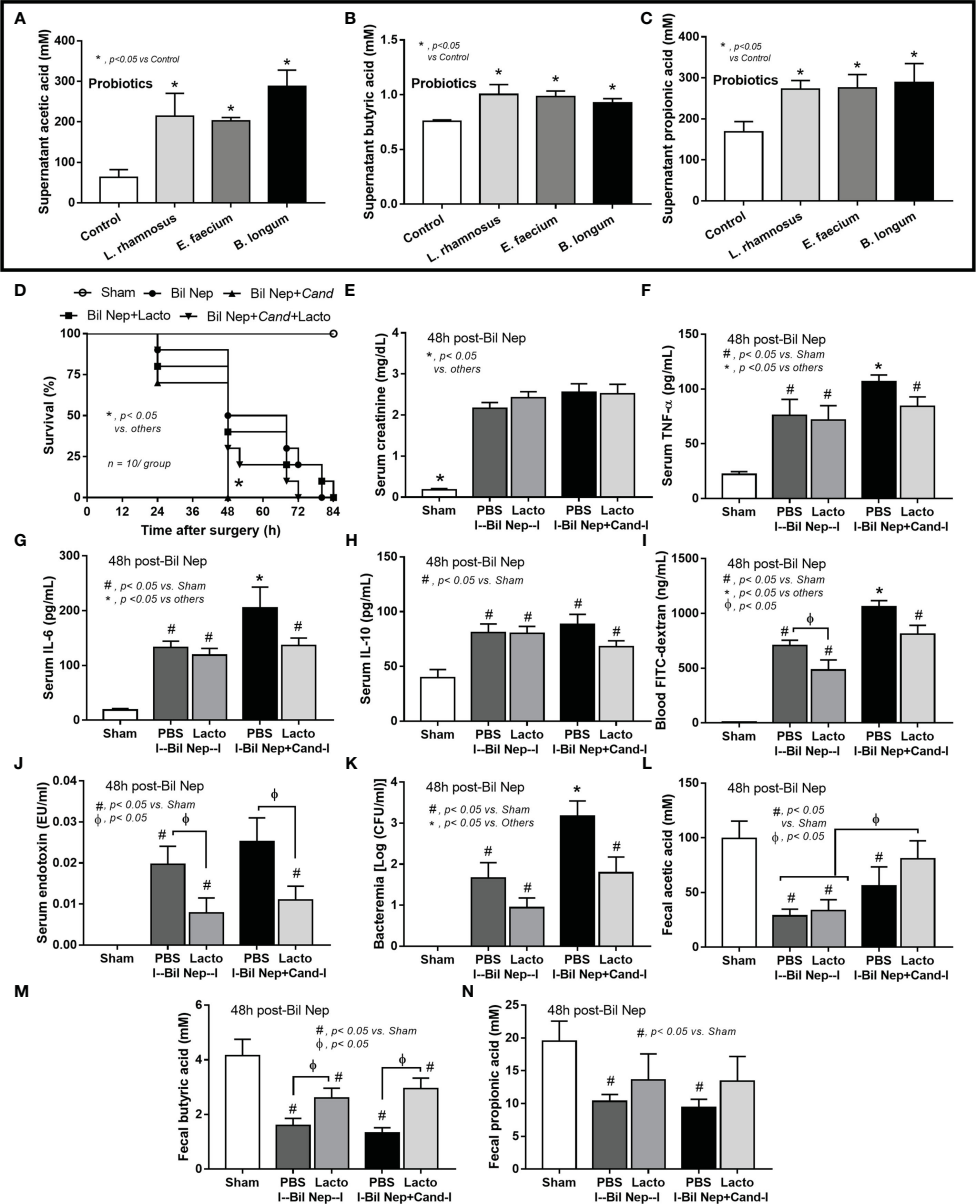
The short chain fatty acid (SCFA) (acetic, butyric, and propionic acids) in the condition media of several probiotics (*Lacticaseibacillus rhamnosus, Enterococcus faecium*, and *Bifidobacterium longum*) compared with the control media (control) **(A–C)** are demonstrated (independent triplicate experiments were performed). Characteristics of bilateral nephrectomy (Bil Nep) mice with or without *Candida* administration (Cand) after treatment by *Lacticaseibacillus rhamnosus* (Lacto) or normal saline solution (NSS) control versus sham mice as indicated by survival analysis **(D)**, serum creatinine **(E)**, serum cytokines (TNF-α, IL-6, and IL-10) **(F–H)**, and parameters of gut barrier defect (FITC-dextran assay, endotoxemia, bacteremia) (**I–K)**, and fecal SCFA (**L–N**) are demonstrated (n = 10/group for **D** and 6-8/group for **E–N**.

Although the increased SCFAs after probiotics administration might be produced from probiotics or the probiotics-promoted bacteria, SCFAs-producing probiotics ensure the enhanced production of SCFAs. As such, several SCFA-related compounds (acetic, butyric, and propionic acid) were detectable in the condition media of several probiotics from our library, including *Lacticaseibacilli*, *Enterococci*, and *Bifidobacterium*, without the significant differences among probiotics (**Figures 7A-C**). Due to the less difficult preparation processes, *Lacticaseibacilli* were selected to use in the mice. Although all mice were dead within 64 h post-Bil Nep, *Candida*-administered Bil Nep (without probiotics) demonstrated the earliest death as all mice died within 48 h (**Figure 7D**) implying an adverse effect of *Candida* gavage. In comparison with Bil Nep without *Candida*, *Candida*-Bil Nep demonstrated more prominent systemic inflammation (serum cytokines), and leaky gut (FITC-dextran assay and bacteremia but not endotoxemia), despite the similar uremic severity (serum creatinine) and the decreased fecal SCFAs (acetic, butyric, and propionic acid) (**Figures 7E–N**). In parallel, probiotics attenuated leaky gut severity in Bil Nep mice as indicated by FITC-dextran assay and endotoxemia (but not bacteremia) with enhanced fecal butyric acid without an alteration in other parameters (**Figures 7D–N**). In *Candida*-Bil Nep, probiotics improved survival rate, serum IL-6 (not TNF-α and IL-10), endotoxemia, and bacteremia with increased fecal butyric acid (**Figures 7D–N**).

**Figure 8:**
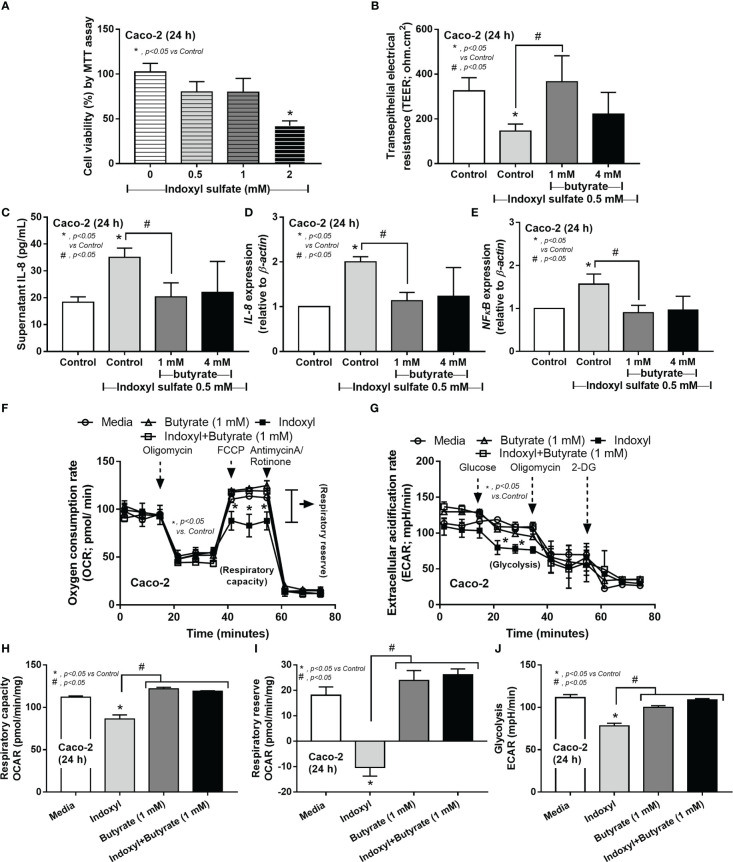
The cell viability (MTT assay) of enterocytes (Caco‐2 cells) after incubation by the different concentrations of indoxyl sulfate **(A)** is demonstrated. Characteristics of Caco-2 cells after incubation by 0.5 mM indoxyl sulfate with butyrate (1 and 4 mM) or control media (Control) as evaluated by transepithelial electrical resistance (TEER) **(B)** and supernatant IL-8 **(C)**, expression of IL-8 and NF-κB **(D, E)** are shown. Additionally, the cell energy status of Caco-2 cells after being activated by 0.5 mM indoxyl sulfate with or without 1 mM butyrate or media control as indicated by indicators for mitochondrial function; oxygen consumption rate (OCR), and glycolysis activity; extracellular acidification rate (ECAR) **(F, G)** with some energy phenotype profile (maximal respiration, respiratory reserve, and glycolysis) **(H–J)** are also demonstrated (independent triplicate experiments were performed for all experiments).

Because i) intestinal secretion of indoxyl sulfate (IS), a gut-derived uremic toxin transforming from indole (tryptophan derivatives of gut-dysbiosis bacteria) at the liver, in uremic conditions is mentioned (51), ii) the enterocyte toxicity of IS (52), and iii) the impact of SCFAs in enterocyte homeostasis (32) and the probiotics-increased fecal butyrate (**Figure 7M**), IS and butyrate were tested in enterocytes. Indeed, enterocyte toxicity of IS was demonstrated by MTT assay with a 50% reduction in cell viability from 2 mM of IS (**Figure 8A**). Despite the non-alteration of cell viability by 0.5 mM IS, this concentration induced enterocyte injury, as indicated by epithelial integrity (TEER), supernatant IL-8, and the upregulated inflammatory genes (*IL-8* and *NFκB*), possibly from the reduction of cell energy (mitochondrial and glycolysis activities) (**Figures 8B–J**). Interestingly, butyrate (at 1 and 4 mM) similarly attenuated enterocyte injury (TEER, supernatant IL-8, *IL-8*, and *NFκB*) with an improvement of cell energy status (**Figures 8B–J**), perhaps through butyrate-related energy supplement (53, 54). In brief, *Lacticaseibacilli* were selected from several SCFA-producing probiotics to use in mice due to the simpler laboratory preparation and the probiotic administration enhanced fecal butyrate (**Figure 7M**) and attenuated the model severity, especially leaky gut, endotoxemia, and bacteremia (**Figure 7I–K**), similarly between *Candida*-Bil Nep and Bil Nep alone. The increased fecal butyrate, either from the administered probiotics or other probiotics-induced beneficial bacteria, partly due to the influence of butyrate on uremic toxin-induced enterocyte damage through enhanced cell integrity (TEER), anti-inflammation, and improved cell energy status (mitochondria and glycolysis activities) (**Figure 8A–J**).

## 4 Discussion

Probiotics attenuated uremia-induced leaky gut in Bil Nep mice with or without *Candida* partly through the short-chain fatty acids (SCFAs)-improved cell energy of enterocytes.

### 4.1 The presence of *Candida* in the gut worsened uremia-induced dysbiosis

The excretion of uremic toxins through the intestine during renal impairment is one of the obvious causes of uremia-induced leaky gut (55), as the direct injury from uremic toxins (such as urea, p-cresol, and indoxyl sulfate) against enterocytes is demonstrated *in vitro* (52, 56, 57), that is possibly an underlying mechanism of enterocyte apoptosis and leaky gut in uremia (2). The toxins (water-soluble and protein-bound compounds) contact intestinal epitheliums through the intestinal lumens (luminal side) and from blood delivery as some toxins (such as indoxyl sulfate) are produced in the blood (through livers) before the delivery to other organs, including kidney, brain, bone, and gut (58), while some toxins (such as p-cresol) are produced within the gut (59). Despite the different production sites, gut-derived uremic toxins (60) are partly responsible for uremia-induced gut dysbiosis, possibly due to the selection of bacteria by the toxins (enhanced bacteria that can utilize the toxins) (61–63) and the direct impacts of uremic toxins on enterocyte damages that also causing dysbiosis (gut inflammation-induced dysbiosis) (64). Here, the presence of *Candida* in the gut caused more severe dysbiosis than Bil Nep alone, as indicated by the increased Proteobacteria (especially Enterobacteria) with the possible direct epithelial invasion by *Candida* spp. (65), which might be correlated with the more severe leaky gut in *Candida*-Bil Nep compared with Bil Nep alone. Indeed, the selection of some bacterial groups by *Candida*, including Enterobacteria and some *Enterobacter* spp., is possibly due to the ability of bacteria to digest the *Candida* cell wall by β-glucanase and cellulolytic enzymes (19, 66). Additionally, the possibly bactericidal activity of some fungal toxins, such as candidalysin (a cytolytic pore-forming peptide), and fungal by-products, such as acetaldehyde, alcohol, and ammonia, also possibly alter some groups of bacteria more than other groups, leading to *Candida*-induced gut dysbiosis (67, 68). In a previous publication, *Candida* administration in sham control mice does not induce gut dysbiosis and systemic inflammation, while *Candida* administration in Bil Nep mice worsens gut dysbiosis and systemic inflammation (2) similar to our current results. Not only in acute uremia, *Candida* administration in chronic kidney disease (CKD) in 5/6 nephrectomy mice also induces gut dysbiosis and worsens systemic inflammation through leaky gut-induced endotoxemia and glucanemia that are severe enough to induce fibrosis in several internal organs (kidney, liver, and heart) approximately at 20-week post-surgery (12, 29). In this aspect, the presence of gut fungi enhances the severity of uremia-induced systemic inflammation partly through leaky gut-induced endotoxemia and glucanemia which might depend on the abundance of Gram-negative bacteria and fungi, respectively, in the gut. The monitoring of leaky gut and the abundance of endotoxin and beta-glucan in gut content might be interesting as biomarkers for monitoring of the disease severity in patients with acute or chronic uremia might be beneficial. More studies on these topics are interesting.

### 4.2 Uremia caused metabolome alteration in feces and in blood with a subtle alteration by *Candida* gavage

Due to the well-known association between gut bacteria and the alteration of small molecules (69), the metabolome analysis was evaluated. As such, the difference in the pattern of fecal metabolome between uremic mice and sham control was obvious, especially the reduction of amino acids, butyrate, and propionate (but not acetate), as indicated by principal component analysis (PCA) and partial least squares-discriminant analysis (PLS-DA) analyses. Perhaps, the enhanced fermentation of proteins in the gut during uremia-induced dysbiosis leads to an increase in amino acids in feces (62, 70) and the reduced normal microbiota from uremia decreases fecal SCFAs (71). Meanwhile, the reduced fecal creatinine in uremic mice compared with sham supported an increase in bacteria that can metabolite creatinine (62, 72). Here, the elevation of several amino acids, except for lysine, aspartate, methionine, and glutamine, supported the increased nonessential amino acids and reduced essential amino acids (histidine, isoleucine, leucine, lysine, methionine, phenylalanine, threonine, tryptophan, and valine) in uremia (73). Indeed, the increase in glutamine and aspartate-associated proteins in uremia are previously mentioned (74, 75). Nevertheless, *Candida* gavage subtly altered uremic fecal metabolome with an increase in acetate and formate compared with uremia alone suggesting the promoted acetate- and formate-producing bacteria (perhaps through the digestion of *Candida* cell wall) (76, 77). In blood metabolome analysis, fewer compounds, possibly only with the high abundance, were detectable with a similar trend of the fecal metabolome, except for taurine and alanine (higher in feces but lower in the blood of uremia compared with sham), and pyruvate (lower in feces but higher in the blood of uremia compared with sham). This fecal-blood discordance of metabolome results indicates a complex correlation of the individual compounds such as the utilization by enterocytes and the transport mechanisms (78). In the blood of uremic mice compared with sham, the reduced 3-hydroxy butyrate (a main ketone body transforming from SCFAs by liver) (79) with the non-difference in acetate and isobutyrate (butyrate isomerization) (80) suggested the difference influences of different SCFAs in uremia. Despite the differences in PLS-DA analysis of blood metabolome between *Candida*-Bil Nep versus Bil Nep, the components in blood metabolome were not different between groups, while some alterations in fecal metabolome were detectable suggesting that *Candida* altered only the metabolites that were possibly too low to be detectable in blood. Despite a similarly low level of fecal SCFAs between Bil Nep versus *Candida*-Bil Nep, *Candida*-Bil Nep demonstrated more severe dysbiosis (the higher abundance of Proteobacteria with the lower bacterial diversity) suggesting that the presence of *Candida* in the gut and the selective gut bacteria from *Candida* had a less impact on fecal SCFAs but demonstrated a higher impact on leaky gut. However, Bil Nep mice with the presence of *Candida* in the gut might be more resemble to the patients due to the higher abundance of fungi in the human gut compared with mouse intestines (27, 28). Because of the higher disease severity of *Candida*-Bil Nep mice over Bil Nep alone, the probiotics that are effective in Bil Nep mice might be ineffective on the *Candida*-Bil Nep group and the test of probiotics on *Candida*-Bil nep mice might be more appropriate for further clinical translation.

### 4.3 Probiotics attenuated uremia-induced leaky gut partly through the improved enterocyte cell energy status by SCFAs

Probiotics attenuate leaky gut from several causes, including acute and chronic uremia, through several mechanisms, including normalized gut dysbiosis, regulation of host immune responses, facilitated mucin production, and induced anti-inflammation by exopolysaccharide and SCFAs (81–83). Among these mechanisms, the production of SCFAs seems to be an interesting mechanism of probiotics as SCFAs, from probiotics themselves or from beneficial bacteria that are promoted by probiotics, are absorbed into enterocytes before using as an energy source (84) with induction of several anti-inflammatory signals (in enterocytes and immune cells) (85). Although the increased intestinal SCFAs after probiotic administration is well-known (86), data on probiotics-enhanced SCFAs in uremia is still less. In a healthy condition, normal gut microbiota enables the transformation of complex nutrients, including plant cell wall components, into simple sugars that are fermented to form SCFAs, mainly formate, acetate, propionate, and butyrate as contributed to more than 90% of SCFAs in the human gut (87, 88). With the reduced SCFAs, several probiotic bacteria can produce SCFAs, including *Lacticaseibacillus rhamnosus* GG*, Bifidobacterium longum* SP 07/3, and *Enterococcus faecalis* AG5 (83, 89). Although the non-SCFAs-producing probiotics can enhance intestinal SCFAs through the promotion of other beneficial bacteria in the gut, the use of SCFAs-producing bacteria possibly ensures SCFAs production. Despite several isolated probiotics with effectively produced SCFAs in our facility, *Lacticaseibacillus rhamnosus* dfa1 was selected to use due to the easier preparation process of the facultative anaerobes compared with the strictly anaerobic Bifidobacterium preparation. Indeed, the *Lacticaseibacilli* attenuated leaky gut and systemic inflammation with increased butyrate in uremia mice with or without *Candida*. Because of the low fecal butyrate both in feces and in blood of uremic mice here, the enhanced butyrate in feces might be a probiotic-activity biomarker as a previous report of low butyrate in uremia patients (90). However, uremia did not alter acetate (the most abundant SCFA) but reduced propionate only in feces (not in blood). Hence, patients with uremia with low fecal butyrate might be more benefit from the administration of SCFAs or probiotics-induced SCFAs and treatment monitoring of the probiotic effects in these patients through fecal metabolome changes (increased fecal butyrate when compared with the before treatment) might be useful. Notably, the treatment monitoring of probiotics effect by blood metabolome measurement might be less beneficial than fecal metabolome evaluation as a subtle difference in blood metabolome between Bil Nep mice with versus without probiotics.

During uremia, several uremic toxins induced damage to enterocytes as serum from patients with chronic kidney disease, urea, and some gut-derived uremic toxins reduced enterocyte integrity (transepithelial electrical resistance; TEER) (91, 92). Here, indoxyl sulfate, a gut-derived uremic toxin was used as a representative toxin for the *in vitro* test in enterocyte cell lines. As such, an impact of butyrate on uremic toxin (indoxyl sulfate)-stimulated enterocytes was demonstrated *in vitro* (enterocyte integrity and anti-inflammation) along with the increased enterocyte cell energy supporting a previous publication (93). The administration of butyrate as a chemical drug is interesting due to an easier production process than probiotic preparation; however, the proper dose adjustment will be necessary as butyrate toxicity is possible (94, 95). Although the more profound enhancement of mitochondrial functions over glycolysis by SCFAs (96, 97) and the increased cell status by butyrate as a source of acetyl coenzyme A (acetyl Co-A) in mitochondrial tricarboxylic acid (TCA) cycle (53) are previously reported, butyrate could not alter cell energy status in the control enterocytes here. On the other hand, indoxyl sulfate (IS) reduced both mitochondrial and glycolysis activity of enterocytes possibly through the enhanced oxidant species (98–100) that were toxic to both mitochondria (oxidant-induced mitochondrial injury) and glycolysis (blockage of glyceraldehyde-3-phosphate dehydrogenase by oxidants) (101). In enterocytes, butyrate normalized IS-induced mitochondrial defect possibly through the increased acetyl Co-A for mitochondrial TCA (102), while improved IS-reduced glycolysis through the improved cell conditions that facilitate glucose utilization (103). Notably, the probiotics were not administered in the sham control because there was no dysbiosis (the imbalance of gut microbiota correlating with the unhealthy condition) in sham mice with the well-known safety of *L. rhamnosus* probiotics (2, 7, 12, 25, 26, 47).

In conclusion, uremia altered metabolome characteristics in both feces, especially reduced butyrate, and the presence of *Candida* in the gut of Bil Nep mice worsened leaky gut-induced systemic inflammation. Despite the enhanced enterocyte damages by *Candida*, probiotics attenuated the model severity, as indicated by increased fecal butyrate, strengthened gut barrier, and reduced leaky gut-induced systemic inflammation. Additionally, the improved fecal butyrate levels after probiotic administration might be a monitoring biomarker for a proper probiotic effect. More studies on these topics are interesting.

## Data availability statement

The datasets presented in this study can be found in online repositories. The names of the repository/repositories and accession number(s) can be found below: https://www.ncbi.nlm.nih.gov/, PRJNA909950.

## Ethics statement

The animal study was reviewed and approved by The animal care and use protocol (SST 028/2564) was certified by the Institutional Animal Care and Use Committee of Chulalongkorn University’s Faculty of Medicine in Bangkok, Thailand, in compliance with the US National Institutes of Health criteria.

## Author contributions

The followings are the authors’ contribution: conceptualization: WC and AL. Methodology: WC, SK, PV, SS, TC, NS, SS, and AL. Validation: SS, TC, SS, PV, and AL. Formal analysis: WC, SK, and AL. Investigation: NS, TC, PV, WC, and AL. Resources: WC, SK, and AL. Data curation: AL. Writing-original draft preparation: WC and AL. Writing review and editing: WC and AL. Supervision: AL. And funding acquisition: WC. All authors contributed to the article and approved the submitted version.
